# Coagulation Dysfunction in Acute Respiratory Distress Syndrome and Its Potential Impact in Inflammatory Subphenotypes

**DOI:** 10.3389/fmed.2021.723217

**Published:** 2021-08-20

**Authors:** Samantha A. Livingstone, Karin S. Wildi, Heidi J. Dalton, Asad Usman, Katrina K. Ki, Margaret R. Passmore, Gianluigi Li Bassi, Jacky Y. Suen, John F. Fraser

**Affiliations:** ^1^Critical Care Research Group, The Prince Charles Hospital, Brisbane, QLD, Australia; ^2^Faculty of Medicine, The University of Queensland, Brisbane, QLD, Australia; ^3^Cardiovascular Research Institute Basel (CRIB), Basel, Switzerland; ^4^Inova Fairfax Hospital, Falls Church, VA, United States; ^5^Department of Anesthesiology and Critical Care, The University of Pennsylvania, Philadelphia, PA, United States; ^6^Department of Pulmonology and Critical Care, Hospital Clínic de Barcelona, Universitad de Barcelona and IDIBAPS, CIBERES, Barcelona, Spain

**Keywords:** acute respiratory distress syndrome, coagulation, anticoagulation, subphenotypes, inflammation

## Abstract

The Acute Respiratory Distress Syndrome (ARDS) has caused innumerable deaths worldwide since its initial description over five decades ago. Population-based estimates of ARDS vary from 1 to 86 cases per 100,000, with the highest rates reported in Australia and the United States. This syndrome is characterised by a breakdown of the pulmonary alveolo-epithelial barrier with subsequent severe hypoxaemia and disturbances in pulmonary mechanics. The underlying pathophysiology of this syndrome is a severe inflammatory reaction and associated local and systemic coagulation dysfunction that leads to pulmonary and systemic damage, ultimately causing death in up to 40% of patients. Since inflammation and coagulation are inextricably linked throughout evolution, it is biological folly to assess the two systems in isolation when investigating the underlying molecular mechanisms of coagulation dysfunction in ARDS. Although the body possesses potent endogenous systems to regulate coagulation, these become dysregulated and no longer optimally functional during the acute phase of ARDS, further perpetuating coagulation, inflammation and cell damage. The inflammatory ARDS subphenotypes address inflammatory differences but neglect the equally important coagulation pathway. A holistic understanding of this syndrome and its subphenotypes will improve our understanding of underlying mechanisms that then drive translation into diagnostic testing, treatments, and improve patient outcomes.

## Introduction

Acute Respiratory Distress Syndrome (ARDS) is a critical respiratory syndrome plagued by severe hypoxaemia and loss of pulmonary function (1). Five decades after it was first described (2) ARDS still represents over 10% of all Intensive Care Unit (ICU) admissions and is the cause of almost one quarter of patients requiring mechanical ventilation. It is also responsible for hundreds of thousands of deaths worldwide annually (3).

ARDS pathophysiology is characterised by dysregulated inflammation and associated coagulation dysfunction which can result in multisystem organ failure. In particular, ARDS severely affects the lungs leading to severe tissue damage, flooding of alveoli with proteinaceous fluid, decreased pulmonary function (4), and ultimately death in ~40% of patients (1, 3, 4). Thus far, disease-modifying treatments in ARDS have been largely unsuccessful (5) and current management strategies focus on supportive approaches to improve oxygenation and reduce lung injury stretch. Coagulation dysfunction in ARDS is a well-recognised phenomenon, largely due to exposure of tissue factor (TF) (6) with subsequent activation of the coagulation pathways, and loss of endogenous anticoagulant function. This leads to unregulated intravascular coagulation, microvascular and endothelial damage, and ultimately the dysfunction of essential organs such as the lungs, heart and kidneys (3, 4). The variability in severity of dysregulation and inconsistency in response to treatment suggest that a single underlying mechanism is very unlikely (6).

In recent years, several retrospective landmark analyses have proven the presence of two inflammatory ARDS subphenotypes, differentiated by inflammatory markers, and biological and physiological parameters (7–11). Thus far, the ARDS inflammatory subphenotypes mainly focus on the inflammation pathways, largely overlooking the equally important coagulation pathway. This article aims to address this important coagulation side of ARDS by discussing prior knowledge of coagulation dysfunction, its interaction with inflammation, and the likely role in ARDS inflammatory subphenotypes.

## Definition and Current Knowledge of ARDS

ARDS was first described by Ashbaugh et al. in 1967. As knowledge on this syndrome grew over the next five decades, new definitions arose to improve rapid recognition and therefore time to diagnosis (Supplementary Table 1, Online Supplement). The Berlin Definition is most commonly used clinically since established in 2012 (12). It defines ARDS as an acute, diffuse, inflammatory lung injury, leading to increased pulmonary vascular permeability, pulmonary oedema, and loss of aerated tissue with consecutive hypoxaemia and bilateral radiographic opacities, associated with venous admixture, increased physiological dead space, and decreased lung compliance (12). The Berlin Definition further categorises patients as mild, moderate and severe ARDS based on their PaO_2_/FiO_2_ ratio (PF ratio) which correlates closely with mortality (12) (Supplementary Table 2, Online Supplement).

Regardless of the cause of ARDS, this syndrome has a high mortality rate of ~40% (1, 3) (Supplementary Table 2, Online Supplement). Patients are still treated in a homogeneous fashion with supportive management, prone positioning (13), and a focus on lung protective mechanical ventilation (14, 15); the only identified key measures known to significantly reduce mortality; outside the use of extracorporeal membrane oxygenation in severe cases (16).

## The Coagulation Cascade

**Figure 1:**
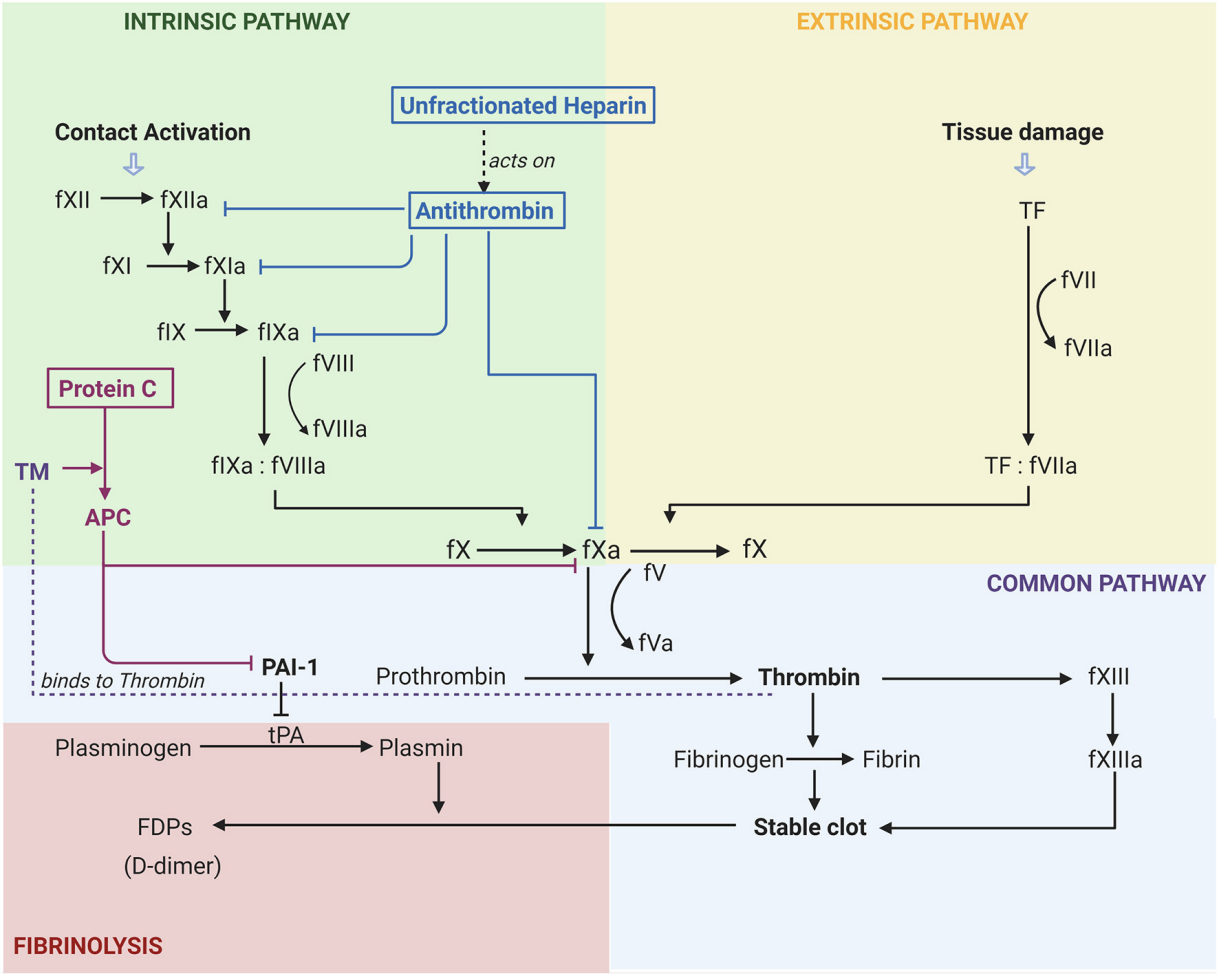
Activation of coagulation pathways and the effects of common anticoagulants in Acute Respiratory Distress Syndrome. F(number)a, factor, “a” indicates active; TF, tissue factor; FDP, Fibrinogen Degradation Product; tPA, Tissue Plasminogen Activator; PAI-1, Plasminogen Activator Inhibitor 1; TM, Thromobomodulin; APC, Activated Protein C. The activation of the extrinsic pathway (yellow) occurs due to tissue damage, whilst the activation of the intrinsic pathway (green) occurs with contact activation. Both of these lead to the common coagulation pathway (purple) and ultimately the fibrinolytic pathway (red). It can be seen that unfractionated heparin acts on antithrombin to inhibit coagulation factors XII, XI, IX, and X. Thrombomodulin is essential for the activation of protein C, and the subsequent inhibition of PAI-1. Figure created with BioRender.com.

The coagulation cascade is made up of the intrinsic and extrinsic pathways that diverge on the common pathway. As seen in Figure 1, the intrinsic pathway is a chain reaction of one factor becoming activated and leading to the activation of the next coagulation factor. In this pathway factors XII, XI, and IX become active leading to fIXa complexing with fVIIIa which then contributes to the activation of fX (17). On the other hand, tissue damage leads to the activation of the extrinsic pathway where TF complexes with fVIIa leading to further activation of fX. fX is where the two pathways merge into the common pathway (17). It is in this common pathway that the prothrombinase complex (fXa:fVa) catalyses the conversion of fII to fIIa, also known as thrombin (17).

## Pathophysiology of ARDS and Associated Coagulation Dysfunction

**Figure 2:**
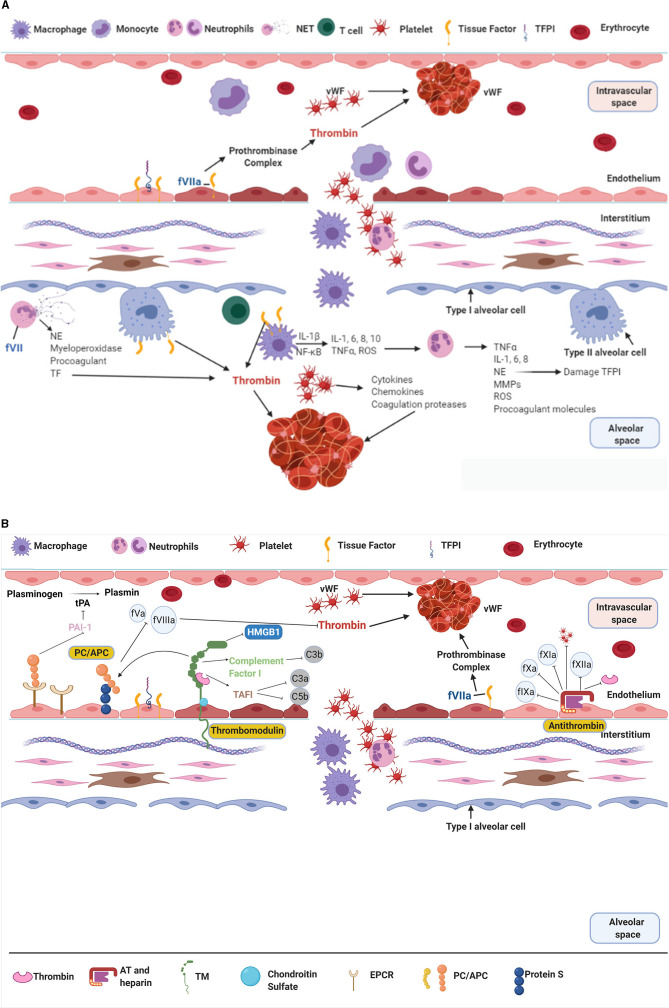
**(A,B)** The interaction of inflammation and coagulation in ARDS. NET, Neutrophil Extracellular Trap; vWF, von Willebrand Factor; TFPI, Tissue Factor Pathway Inhibitor; IL-, Interleukin; NE, Neutrophil Elastase; ROS, Reactive Oxygen Species; TNFα, Tumour Necrosis Factor α; MMP, Matrix Metalloproteinases; TF, Tissue Factor; tPA, tissue Plasminogen Activator; PAI-1, Plasminogen Activator Inhibitor-1; HMGB1, High Mobility Group Box protein 1; C3/5 a/b, Complement factor; TAFI, Thrombin Activatable Fibrinolysis Inhibitor; PC, Protein C; TM, Thrombomodulin; EPCR, Endothelial Protein C Receptor; AT, Antithrombin. The role of coagulation and inflammation are intrinsically linked together, both occurring in parallel in ARDS. Ultimately their dysfunction leads to the damage seen in ARDS patients. **(A)** The endogenous anticoagulants PC, TM, and AT are responsible for controlling coagulation. These proteins respond to a procoagulant/pro-inflammatory environment by reducing producing of thrombi and promoting fibrinolysis. **(B)** In ARDS the activation of macrophages and neutrophils leads to the upregulation of inflammation and coagulation. There is production of interleukins, TNFα, reactive oxygen species, etc that lead to damage to the epithelial and endothelial surface. This allows further movement of inflammatory cells and protein rich fluid into the alveoli, leading to alveolar collapse and loss of ventilatory units. The upregulation of inflammation with ongoing tissue damage is closely matched by the activation of the coagulation system, and production of thrombi. Figure created with BioRender.com.

Although there are multiple risk factors that can lead to the development of ARDS, spanning from sepsis and pneumonia to trauma and transfusions (4); in terms of pathophysiology, these injuries lead to a stereotypic response to the initial event (initial exudative phase) characterised by damage of the endothelial and/or epithelial barrier (4). This activates the innate immune system (6), with alveolar macrophages and neutrophils all contributing to a pro-inflammatory state through the release of cytokines (i.e., tumour necrosis factor α (TNFα), interleukin 1, 6, and 8 (IL-1, IL-6, and IL-8) and inflammatory mediators (i.e., monocyte chemoattractant protein 1, reactive oxygen species) (20–23). The damage to the endothelial cells and upregulation of the inflammatory system leads to increased levels of microvesicles (24) and inflammatory cells expressing TF, along with activation of the coagulation system. TF can be expressed from microvesicles, macrophages, activated epithelial cells and endothelial cells, which then bind to fVII and stimulate the coagulation pathway (6, 25). Additionally, neutrophils and neutrophil extracellular traps release neutrophil elastase (NE), which causes further damage to the endothelial cytoskeleton and alveolar capillary barrier and contribute to coagulation by the release of procoagulants and TF (6). The activation of the coagulation pathway and upregulation of inflammatory mediators gives way to migration and activation of platelets (Figure 2A). In response platelets activate, change shape and release further inflammatory and coagulation markers, as well as interact with neutrophils, leading to platelet aggregation and activation, and clot formation (6).

The anticoagulation control system [protein C (PC), thrombomodulin (TM) and antithrombin (AT)] are designed to regulate the balance between coagulation and anti- coagulation within the body and its detrimental effects (Figure 2B). Consequently, in proinflammatory states these mechanisms become damaged leading to dysregulated haemostasis. Subsequently, the activated coagulation pathways lead to mass thrombin formation, adding to obstruction of the microcirculation (20, 26, 27). The resulting decreased total luminal diameter of the vascular bed can lead to pulmonary hypertension with subsequent deterioration in right ventricular function (28, 29), as well collateral tissue damage and organ dysfunction (6).

An important regulator of endogenous anticoagulant function and a mediator between inflammation and coagulation comes from a family of G-coupled protein receptors known as Protease Activated Receptors (PARs) (6, 30). When activated by peptidases via cleavage of the extracellular N-terminal domain, PARs cause upregulation of cytokines, inflammatory mediators, and platelet aggregation (20, 31). For example, when PAR-1 is activated by activated PC or low levels of thrombin it exerts a cytoprotective function; but in the presence of high thrombin levels the effect is the opposite by contributing to damage of the endothelial barrier (6). These receptors act like biological switches, varying their effect based on the environment, and the levels of PC and thrombin present.

## Endogenous Anticoagulants in ARDS

There are three main potent endogenous anticoagulant systems that occur to prevent unregulated coagulation and, to some degree, inflammation in patients (Figure 2B). The multi-factorial role (inflammation and coagulation) of these endogenous anticoagulants makes them key areas of investigation in ARDS subphenotypes (32–38).

### Protein C

PC circulates in healthy individuals as an inactive zymogen and is modified to Activated Protein C (APC) in the presence of TM and Thrombin. APC has potent anticoagulant effects as a result of proteolytic degradation of fV and fVIII (6). In addition, it also possesses anti-inflammatory qualities via PAR-1 by inhibiting the Nuclear Factor-κB pathway; and pro-fibrinolytic properties by inhibiting plasminogen activator inhibitor-1 (PAI-1) (6, 32). The action of APC with endothelial protein C receptor (EPCR) on PAR-1 leads to endothelial protection and stabilising by activating the spingosine-1-phosphate receptor 1 (39, 40). APC/EPCR also act on PAR-2 to shift from a barrier disruptive to a barrier protective effect in endothelial cells (41), both examples of how PC is essential not just for coagulation control, but also inflammation. In ARDS patients, PC and platelet levels are reduced and PAI-1 levels subsequently augmented, and these changes have been correlated with a procoagulant/antifibrinolytic state and increased mortality (34, 42). Interestingly, although mortality rates were not significantly altered, recombinant APC (rAPC) administration has been shown to reduce intrapulmonary coagulation disturbances, stimulate fibrinolysis and improve oxygenation in ovine and murine sepsis models (38, 43–45). Unfortunately, the use of rAPC in sepsis patients in a clinical trial setting was discontinued after the 2012 PROWESS-SHOCK trial failed to show a significant difference in mortality (46). In a phase II trial investigating the use of rAPC in patients with Acute Lung Injury (no sepsis) there was no difference between the treatment group and placebo in regards to ventilator free days and 60 day mortality (47). The failure of rAPC to show significant improvements in mortality in heterogenous patient clinical trials question if smaller, more specific phenotypic subpopulations would respond more favourably to APC, but this remains unknown. The reduced levels of circulating PC, and increased levels of PAI-1 in human hyperinflammatory subphenotypes may be an indicator that PC is substantially more affected by a hyperinflammatory state. Additionally, the anticoagulant, anti-inflammatory and pro-fibrinolytic properties of APC make it a protein of likely significance in ARDS subphenotypes.

### Thrombomodulin

TM, a transmembrane endothelial protein, is a potent anticoagulant that binds and alters thrombin's function from procoagulant and pro-inflammatory to an anticoagulant and anti-inflammatory state (26, 48), activates PC, as well as Thrombin Activatable Fibrinolysis Inhibitor which inactivates complement derived anaphylatoxins C3a and C5a, bradykinin and osteopontin (49). TM is also an anti-inflammatory agent via activation of PC (50–53), attenuation of high mobility group box protein 1 (HMGB1) (54) and enhancement of complement factor I with subsequent inactivation of C3b (49, 51, 52, 55, 56). The attenuation of HMGB1 causes suppression of pathogen associated molecular patterns and damage associated molecular patterns, leading to dampening of their interaction with cell surface receptors on innate immune cells generating a marked downregulation in inflammatory upregulation (55).

A small portion of TM exists in a soluble form (sTM) in plasma, around 3–50 ng/mL under normal conditions (56). sTM is caused by neutrophil derived enzymatic cleavage of the extracellular portion of TM, and these soluble forms vary in length, only retain around 30–50% of their cofactor activity and have reduced anti-inflammatory activity (55, 57, 58). TM levels vary in different diseases, with thrombin, vascular endothelial grown factor, heat shock proteins, and histamines upregulating TM gene expression; and shear stress, haemodynamic forces, transforming growth factor-β, hypoxia and oxidised low density lipoproteins causing downregulation (56). In addition, the expression of TM can be reduced by TNFα, IL-1β, and endotoxin, which cause proteolytic cleavage, internalization, and suppression of transcription (56). In proinflammatory conditions, such as those found in sepsis and ARDS cytokine release causes internalisation and reduced expression of TM, along with leukocyte protease cleavage of TM from the membrane and subsequent increased levels of poorly functional sTM (32, 55, 56). The presence of increased sTM levels in ARDS patients has been positively correlated with cell damage, organ failure, ventilator free days and mortality; as well as distinguishing survivors from non-survivors (42). NE, endotoxin and TNFα have been found to proteolytically cleave small, degraded fragments of sTM from the membrane leading to sTM that have reduced procoagulant and anti-inflammatory properties (42). This indicates that a condition characterised by a hyperinflammatory state may have a greater degree of TM cleavage, leading to increased sTM levels and decreased function.

The human recombinant soluble form of TM (rTM), comprising of the ectodomain of TM has been shown to improve the PF ratio, and reduce ICU stays, as well as the in-hospital and 90-day mortality rates (59) in ARDS patients with concurrent disseminated intravascular coagulation. When used in combination with a potent inhibitor of neutrophil elastase, such as the drug Sivelestat, patients showed significantly less days on mechanical ventilation, increased PF ratios and higher 60-day survival (60). This reinforces the importance of endothelial damage in the pathophysiology of ARDS. As a rate limiting step in the activation of PC, a potent anticoagulant and anti-inflammatory, TM holds great promise in mechanism of action and as a treatment in ARDS subphenotype populations. Ongoing research in this area is currently underway and likely to yield promising results for ARDS patients.

### Antithrombin

AT exerts strong anticoagulant function by inhibiting thrombin, fIXa, fXa, fXIa, fXIIa, plasmin, trypsin, and kallikrein (32, 61). AT covalently binding to glycosaminoglycans on the endothelial barrier also promotes prostaglandin I_2_ formation leading to impairment of cytokine production, activation of leukocytes, and contributes to inhibition of platelet aggregation (62–64). Furthermore, the formation of thrombin-antithrombin complexes leads to the inhibition of thrombin and its pro-inflammatory effects (32). In healthy people there are relatively low concentrations of AT in the lungs (34), but lower free levels are present in ARDS patients likely due to increased consumption of AT which has attenuated the high levels of thrombin present (34, 65). In animal smoke inhalation ARDS models, intravenous infusions of recombinant human AT (rhAT) have led to improved PF ratios, reduced shunt fraction and pulmonary permeability, and reduced neutrophil infiltration to the lungs (62). However, these effects were significantly better in the group that received intravenous rhAT with nebulised heparin and Tissue Plasminogen Activator (tPA) (66). The combination of AT and heparin provide local anticoagulant effects and prevent the formation of casts; and the addition of tPA dissolves already formed fibrin clots and cast structures creating a dual approach to thrombin formation in these models (66).

## ARDS Subphenotypes and Initial Coagulation Dysfunction

Until recently a commonly accepted theory was that all ARDS patients have a similar underlying pathophysiology and therefore treatments were frequently targeted at a heterogenous population. Unfortunately, this is not the case, as evidenced by the lack of efficacious treatment that works for all patients. Recently analyses of previous randomised controlled trials (incorporating the cohorts ARMA, ALVEOLI, FACTT, SAILS, HARP, and MARS) indicate that biological patterns arise within the diverse ARDS population. These retrospective analyses have identified two distinct subgroups, named the hypoinflammatory (P1) and the hyperinflammatory subphenotype (P2), according to distinct functional and biological parameters (7–11, 18, 19). Reports of ARDS subphenotypes have shown P2 was consistently characterised by higher levels of inflammatory cytokines [IL-6, IL-8, IL-10, soluble Tissue Necrosis Factor receptor-1 (sTNFr-1)] and a pronounced shock state with more severe metabolic acidosis (7–10) (Supplementary Table 3, Online Supplement). Looking at the differentiating biomarkers, there is preliminary evidence for a more procoagulant and antifibrinolytic state in P2 (7–10). Furthermore, in all these retrospective analysis the P2 subphenotype had a markedly higher mortality rate than P1, as well as a different reaction to medication intervention (7, 10) or anti-inflammatory treatment (8, 11) (Supplementary Table 3, Online Supplement). This indicates ARDS management may be subphenotype dependent (7, 8, 10).

When examining the limited coagulation parameters available for these subphenotypes, analysis shows that PAI-1 and von Willebrand Factor (vWF) are consistently elevated in P2 patients, while PC and platelets are decreased in P2 patients as compared to P1 patients (7–10). The higher platelet count and protein C levels in P1 could indicate that there is less thrombin formation, therefore less consumption of these key players in coagulation. In contrast, in P2 there could be an increased clot formation along with reduced fibrinolysis due to increased levels of the fibrinolysis inhibitor PAI-1. Bos, Schouten (18) have shown in the MARS cohort that P1 had higher levels of antithrombin, and reduced levels of D-dimer and tissue plasminogen activator. The increased levels of antithrombin suggest there is less consumption of this essential protein, likely due to reduced thrombin levels. However, the increased d-dimer and tPA in light of higher PAI-1 (potent inhibitor of tPA) levels in P2 need further investigation in regard to the balance between these two to create a better understanding of the fibrinolysis cascade. These results reinforce the already known close relationship between inflammation and coagulation, leading to the assumption that the hyperinflammatory subphenotype is more likely to suffer from a procoagulant state, which would favour thrombi formation and subsequent damage such as multi-organ dysfunction. However, in order to provide targeted treatments to patients, a more holistic understanding of how the two subphenotypes vary in terms of their specific coagulation profiles is required. Whilst suspected mechanisms include changes in gene upregulation secondary to a proinflammatory environment and loss of the activated PC precursor TM, which has increased shedding from the endothelial membrane in the presence of proinflammatory states, further research is required to accurately identify these mechanisms and potential treatment targets.

## Discussion

The impact of reduced levels of circulating endogenous anticoagulants (i.e., APC, TM, AT) in ARDS patients appears to have a significant impact in the pathophysiology of this syndrome (34, 42, 67, 68). Studies investigating recombinant forms of these anticoagulants have shown promising results in various populations with improved pulmonary mechanics and reduced hospital stays (34, 36–38, 59, 60, 62, 69). With evidence of two different subphenotypes among the heterogenous population of ARDS patients growing, a serious limitation of studies thus far seems to be the heterogeneity of the studied population, potentially leading to the dilution of a potential treatment and intervention effect.

The consistent identification of differentially expressed parameters of coagulation between the P2 and P1 among the 6 cohorts and the known close relationship between inflammation and coagulation leads to the assumption that the coagulation profiles are indeed different between the ARDS subphenotypes. Further research into classifying the differences in coagulation profiles of these two inflammatory subphenotypes holds potential to show a deeper understanding of the underlying mechanisms driving the subphenotypes, but also may lead to biomarkers for diagnostics and monitoring, as well as targeted anticoagulant treatment tailored to each patient. The treatment of phenotypes of diseases has been an emerging and promising field in cancer, asthma and sepsis to name a few (70). Rather than treating symptoms defining the syndrome, but understanding the underlying biology in more depth, treatments could be targeted to a specific dysfunction, coagulation potentially one of those. Targeting anticoagulant treatment at a more homogenous population based on coagulation profiles of ARDS inflammatory subphenotypes could allow tailored treatment for a patient, potentially reducing prothrombotic-related morbidity.

## Conclusion

ARDS is a critical and debilitating disease, plagued by dysregulated inflammation and coagulation. These abnormalities drive the formation of vascular thrombi and subsequent organ damage, contributing to the high mortality rates of ARDS. Having a greater understanding of how coagulation dysfunction varies in the two ARDS subphenotypes, and how this can be targeted is of great value in creating a holistic understanding and pinpointing therapeutic targets. Future directions should focus on phenotypical subpopulations of ARDS and reducing heterogeneity within patient cohorts which will help increase the likelihood of successful outcomes in treatment trials.

## Author Contributions

SL and KW wrote and prepared the manuscript for publication. AU, HD, GL, JS, JF, KK, and MP assisted in reviewing and preparing the manuscript for publication. All authors contributed to the article and approved the submitted version.

## Conflict of Interest

The authors declare that the research was conducted in the absence of any commercial or financial relationships that could be construed as a potential conflict of interest.

## Publisher's Note

All claims expressed in this article are solely those of the authors and do not necessarily represent those of their affiliated organizations, or those of the publisher, the editors and the reviewers. Any product that may be evaluated in this article, or claim that may be made by its manufacturer, is not guaranteed or endorsed by the publisher.

## Abbreviations

AECC: American European Consensus Conference
ALI: Acute Lung Injury
ANG 1/2: Angiopoietin 1/2
APC: Activated Protein C
ARDS: Acute Respiratory Distress Syndrome
AT: Antithrombin
CPAP: Continuous positive airway pressure
CT: Computed Tomography
CXR: Chest radiography
EPCR: Endothelial Protein C Receptor
f: coagulation factor. An “a” after the factor indicates activated.
FDP: Fibrinogen Degradation Products
HMGB1: High mobility group box protein 1
ICAM-1: Intercellular adhesion molecular-1
ICU: Intensive Care Unit
IFN-y: Interferon gamma
IL: Interleukin
IPPV: Intermittent Positive Pressure Ventilation
MMP: Matrix metalloproteinases
NE: Neutrophil Elastase
NET: Neutrophil Extracellular Traps
PAI-1: Plasminogen Activator Inhibitor-1
PAR: Protease Activating Receptor
PAWP: Pulmonary artery wedge pressure
PEEP: Positive End Expiratory Pressure
PF ratio: PaO_2_/FiO_2_
PC: Protein C
P1: Hypo-inflammatory subphenotype of ARDS
P2: Hyperinflammatory subphenotype of ARDS
RAGE: Receptor for advanced glycation end-products
rAPC: Recombinant activation protein C
rhAT: Recombinant human Antithrombin
ROS: Reactive oxygen species
rTM: Recombinant Thrombomodulin
sTM: soluble Thrombomodulin
TM: Thrombomodulin
TAFI: Thrombin Activatable Fibrinolysis Inhibitor
TF: Tissue Factor
TFPI: Tissue Factor Pathway Inhibitor
TNFα: Tumour Necrosis Factor α
tPA: Tissue Plasminogen Activator
vWF: Von Willebrand factor
WCC: White cell count.
