# Supplementary material for: Coagulation Dysfunction in Acute Respiratory Distress Syndrome and Its Potential Impact in Inflammatory Subphenotypes
Source: Front Med (Lausanne). 2021 Aug 20;8:723217. doi: 10.3389/fmed.2021.723217 (PMC8417599; doi:10.3389/fmed.2021.723217)
Supplement: Supplementary file 1 [file Data_Sheet_1.pdf]

Online Supplement

eTable 1: Definitions of Acute Respiratory Distress Syndrome over time

	Ashbaugh 1967	AECC 1994	Berlin Definition 2012
**Timing**	Nil	Acute	Acute: Within 1 week of known clinical insult or new or worsening respiratory symptoms
**Chest imaging**	Chest radiographs: Diffuse alveolar infiltration	Bilateral infiltrates on frontal chest radiographs	Chest radiographs/CT: bilateral opacities consistent with pulmonary oedema
**Oxygenation**	Not clearly defined, but did report cyanosis refractory to nasal oxygen and IPPV	ALI: PaO_2/FiO_2 ≤300mmHg<br>ARDS: PaO_2/FiO_2 ≤200mmHg<br>*regardless of PEEP*	ALI removed<br><u>Mild</u>: 200mmHg <PaO_2/FiO_2(2) ≤ 300mmHg (CPAP or PEEP ≥5cm H_2O<br><u>Moderate</u>: 100mmHg < PaO_2/FiO_2 ≤200mmHg (PEEP ≥ 5cm H_2O)<br><u>Severe</u>: PaO_2/FiO_2 ≤ 100mmHg (PEEP ≥5cm H_2O)
**PAWP/Oedema**	Nil	≤18mmHg or no evidence of left atrial hypertension	Excluded from definition. Replaced with respiratory failure not fully explained by cardiac failure or fluid overload

Abbrev: AECC: American European Consensus Conference; CT: computer tomography; IPPV: intermittent positive-pressure ventilation; ALI: acute lung injury; ARDS: acute respiratory distress syndrome; PEEP: positive end-expiratory pressure; CPAP: continuous positive airway pressure; PAWP: pulmonary artery wedge pressure

References: (1–3)

eTable 2: Prevalence and mortality of ARDS according to severity

Severity	Prevalence (%)	ICU/hospital mortality (%)
**Mild**	30	34.9
**Moderate**	46.6	40.3
**Severe**	23.4	46.1

Legend: Values are represented in percentages of the ARDS population.

Reference: (4)

eTable 3: Differentiating factors between the two ARDS inflammatory subphenotypes

**Parameters**	**Differentiating markers between subphenotypes**	**Suphenotype levels**	
		P1 levels with respect to P2	
**Laboratory markers**	IL-6, -8, -10, IFN-y	↓	↑
	sTNFr-1, ICAM-1	↓	↑
	Ang-1/-2, RAGE	↓	↑
	WCC	↓	↑
	Creatinine,	↓	↑
	bicarbonate, PaCO2	↑	↓
	albumin, glucose	↑	↓
**Clinical parameters**	Heart rate	↓	↑
	Systolic BP	↑	↓
	Vasoactive use		
	Minute ventilation,	↓	↑
	plateau pressure	↓	↑
	PEEP	↓	↑
	Respiratory rate	↓	↑
		↓	↑
**Coagulation markers**	Protein C	↑	↓
	Platelet count	↑	↓
	PAI-1	↓	↑
	vWF	↓	↑

Abbreviations: IL: interleukin; sTNFr-1: soluble tumor necrosis factor receptor-1; Ang-1/2: angiopoietin-1/2; RAGE: receptor for advanced glycation end-products; CRP: C reactive protein; WCC: white cell count; PCT: procalcitonin; PAI-1: plasminogen activator inhibitor-1; vWF: von willebrand factor; PEEP: positive end expiratory pressure; IFN-y: interferon gamma; ICAM-1: Intercellular adhesion molecular-1; ICAM-1: intercellular adhesion molecule-1; WCC: White cell count; $PaCO_2$: partial pressure of carbon dioxide in arterial blood; BP: Blood pressure.

References: (5–10)

eTable 4: Mortality and reaction to interventions

Cohort	Intervention	Hypoinflammatory	Hyperinflammatory
**ALVEOLI**	**PEEP strategy**	Mortality 24% with higher PEEP; 16% with low PEEP	Mortality 42% with higher PEEP; 51% with low PEEP.
**FACTT**	**Fluid strategy 90 day mortality rate**	26% mortality with fluid liberal strategy; 18% with fluid conservative strategy.	40% mortality rate with fluid liberal strategy; 50% with fluid conservative strategy.
**HARP**	**Simvastatin (28 day mortality)**	16% mortality when treated with Simvastatin; 17% mortality when given placebo.	32% mortality when treated with Simvastatin; 45% mortality when given placebo.
**SAILS**	**Rosuvastatin**	No evidence for subphenotype specific treatment benefit	
**Kitsios et al**	**Comparison of at risk for ARDS and ARDS**	90 day mortality for ARDS 22%; at risk for ARDS 18%	90 day mortality for ARDS 44%; at risk for ARDS 53%

Abbreviations: PEEP: Positive end expiratory pressure; ARDS: Acute Respiratory Distress Syndrome; ALVEOLI: Assessment of Low tidal Volume and elevated End-expiratory volume to Obviate Lung Injury; FACTT: Fluid and Catheter Treatment Trial; HARP: Hydroxymethylglutaryl-CoA Reductase Inhibition with Simvastatin in Acute Lung Injury to Reduce Pulmonary Dysfunction-2; SAILS: Statins for Acutely Injured Lungs from Sepsis.

References: (5–10)

References

1. Ashbaugh DG, Bigelow DB, Petty TL, Levine BE. Acute respiratory distress in adults. *Lancet (London, England)* (1967) **2**:319–23.

2. Bernard GR, Artigas A, Brigham KL, Carlet J, Falke K, Hudson L, Lamy M, LeGall JR, Morris A, Spragg R. Report of the American-European consensus conference on ARDS: Definitions, mechanisms, relevant outcomes and clinical trial coordination. *Intensive Care Med* (1994) **20**:225–232. doi:10.1007/BF01704707

3. Ranieri VM, Rubenfeld GD, Thompson BT, Ferguson ND, Caldwell E, Fan E, Camporota L, Slutsky AS. Acute respiratory distress syndrome: the Berlin Definition. *JAMA* (2012) **307**:2526–2533. doi:10.1001/jama.2012.5669

4. Bellani G, Laffey JG, Pham T, Fan E, Brochard L, Esteban A, Gattinoni L, van Haren F, Larsson A, McAuley DF, et al. Epidemiology, Patterns of Care, and Mortality for Patients With Acute Respiratory Distress Syndrome in Intensive Care Units in 50 Countries. *JAMA* (2016) **315**:788. doi:10.1001/jama.2016.0291

5. Calfee CS, Delucchi K, Parsons PE, Taylor B, Ware LB, Matthay MA. Latent Class Analysis of ARDS Subphenotypes: Analysis of Data From Two Randomized Controlled Trials. *Lancet Respir Med* (2014) **2**:611–620. doi:10.1016/S2213-2600(14)70097-9.Latent

6. Sinha P, Delucchi K, Thompson B, McAuley DF, Matthay MA, Calfee CS. Latent class analysis of acute respiratory distress syndrome (ARDS): Secondary analysis of the statins for acutely injured lungs from sepsis (SAILS) trial. *Am J Respir Crit Care Med* (2018) **197**:

7. Delucchi K, Famous KR, Ware LB, Parsons PE, Taylor Thompson B, Calfee CS. Stability of ARDS subphenotypes over time in two randomised controlled trials. *Thorax* (2018) **73**:439–445. doi:10.1136/thoraxjnl-2017-211090

8. Famous KR, Delucchi K, Ware LB, Kangelaris KN, Liu KD, Thompson BT, Calfee CS, Network A. Acute Respiratory Distress Syndrome Subphenotypes Respond Differently to Randomized Fluid Management Strategy. *Am J Respir Crit Care Med* (2017) **195**:331–338. doi:10.1164/rccm.201603-0645OC

9. Kitsios GD, Yang L, Manatakis D V., Nouraie M, Evankovich J, Bain W, Dunlap DG, Shah F, Barbash IJ, Rapport SF, et al. Host-Response Subphenotypes Offer Prognostic Enrichment in Patients With or at Risk for Acute Respiratory Distress Syndrome*. *Crit Care Med* (2019) **47**:1724–1734. doi:10.1097/CCM.0000000000004018

10. Sinha P, Delucchi KL, Thompson BT, McAuley DF, Matthay MA, Calfee CS. Latent class analysis of ARDS subphenotypes: a secondary analysis of the statins for acutely injured lungs from sepsis (SAILS) study. *Intensive Care Med* (2018) **44**:1859–1869. doi:10.1007/s00134-018-5378-3
